# Dental Pulp Mesenchymal Stem Cells as a Treatment for Periodontal Disease in Older Adults

**DOI:** 10.1155/2020/8890873

**Published:** 2020-08-18

**Authors:** Beatriz Hernández-Monjaraz, Edelmiro Santiago-Osorio, Edgar Ledesma-Martínez, Itzen Aguiñiga-Sánchez, Norma Angélica Sosa-Hernández, Víctor Manuel Mendoza-Núñez

**Affiliations:** ^1^Research Unit on Gerontology, FES Zaragoza, National Autonomous University of Mexico, 09230 Mexico City, Mexico; ^2^Haematopoiesis and Leukaemia Laboratory, Research Unit on Cell Differentiation and Cancer, FES Zaragoza, National Autonomous University of Mexico, 09230 Mexico City, Mexico

## Abstract

Periodontal disease (PD) is one of the main causes of tooth loss and is related to oxidative stress and chronic inflammation. Although different treatments have been proposed in the past, the vast majority do not regenerate lost tissues. In this sense, the use of dental pulp mesenchymal stem cells (DPMSCs) seems to be an alternative for the regeneration of periodontal bone tissue. A quasi-experimental study was conducted in a sample of 22 adults between 55 and 64 years of age with PD, without uncontrolled systemic chronic diseases. Two groups were formed randomly: (i) experimental group (EG) *n* = 11, with a treatment based on DPMSCs; and a (ii) control group (CG) *n* = 11, without a treatment of DPMSCs. Every participant underwent clinical and radiological evaluations and measurement of bone mineral density (BMD) by tomography. Saliva samples were taken as well, to determine the total concentration of antioxidants, superoxide dismutase (SOD), lipoperoxides, and interleukins (IL), before and 6 months after treatment. All subjects underwent curettage and periodontal surgery, the EG had a collagen scaffold treated with DPMSCs, while the CG only had the collagen scaffold placed. The EG with DPMSCs showed an increase in the BMD of the alveolar bone with a borderline statistical significance (baseline 638.82 ± 181.7 vs. posttreatment 781.26 ± 162.2 HU, *p* = 0.09). Regarding oxidative stress and inflammation markers, salivary SOD levels were significantly higher in EG (baseline 1.49 ± 0.96 vs. 2.14 ± 1.12 U/L posttreatment, *p* < 0.05) meanwhile IL1*β* levels had a decrease (baseline 1001.91 ± 675.5*vs.* posttreatment 722.3 ± 349.4 pg/ml, *p* < 0.05). Our findings suggest that a DPMSCs treatment based on DPMSCs has both an effect on bone regeneration linked to an increased SOD and decreased levels of IL1*β* in aging subjects with PD.

## 1. Introduction

Periodontal disease (PD) is an infectious and inflammatory alteration that affects the supporting tissues of the teeth and, when treatment is not appropriate or adequate, it can cause the loss of these [1]. PD presents an immune and anti-inflammatory response caused by antigenic substances from bacteria in the subgingival biofilm; however, the exacerbated host response is ineffective, and therefore chronic inflammation is maintained [2].

During the acute phase of PD, the presence of bacteria, and especially the lipopolysaccharides in your cell wall, attracts macrophages, leukocytes, and neutrophils to the area of infection. The latter contain enzymes such as NADPH oxidase and myeloperoxidase to produce reactive oxygen species (ROS) that help fight pathogens [3, 4].

Under normal conditions, antioxidant mechanisms protect tissues from damage by ROS secreted by neutrophils. However, if the body's antioxidant capacity is insufficient, oxidative stress (OxS) occurs, which is an imbalance between ROS and antioxidants in favor of the former, causing tissue damage [5].

OxS causes oxidation of important enzymes, stimulates the release of proinflammatory interleukins, lipid peroxidation, and DNA, and protein damage. These mechanisms affect the gingival tissues, the periodontal ligament, the root cementum, and the alveolar bone that support the tooth [6, 7].

A prevalence of PD is estimated at around 11% within the world population [8]. This frequency and its complications increase as age increases. In this sense, the prevalence in people over 40 years is higher than 30%. Among the main alterations of this disease can be mentioned the loss of teeth and chewing problems. This disease can even impact social conditions like decreasing self-esteem and affecting social relationships [9].

The infectious and inflammatory processes of PD are closely related to systemic diseases, such as cardiovascular diseases, diabetes mellitus, arthritis, obesity, and Alzheimer's disease [10–12]. In this respect, both these biological alterations and social repercussions may cause a risk of systemic diseases and an overall life quality reduction of the individual. Thus, it is essential to give timely and effective treatment for PD. A high percentage of adults in the aging process with PD require surgical treatment; nevertheless, the results are not entirely satisfactory because the regeneration of the lost tissue is not achieved [13].

For this reason, new therapeutic alternatives have been proposed, amid them, the grafting of dental pulp mesenchymal stem cells (DPMSCs), have shown successful results in preclinical investigations [14–17], yet there are few clinical human studies [18].

The dental pulp cells originate from the neural crest. During the embryonic period, the interaction between oral ectodermal epithelial cells and mesenchymal stem cells (MSCs) leads to the formation of various structures. First, the enamel organ is formed. Then, the papilla and dental follicle are formed. MSCs give rise to other components of the tooth, such as dentin, pulp, cementum, and the periodontal ligament [19]. DPMSCs are the MSCs that lodge in the papilla at the level of the pulp chamber, where they remain until teeth are exfoliated. Given its characteristics, this cell niche is easily accessible, because its morbidity is limited after harvesting and it is considered a suitable candidate for cell-based tissue engineering strategies. Furthermore, it has a wide expansion rate, the potential to differentiate into cells from multiple cells, organs, systems [20, 21], and without having a malignant phenotype [22].

In addition, it has been shown in preclinical studies that DPMSCs differentiate into cementoblast-like cells, adipocytes, and collagen-forming cells, with the ability to generate cement-like material from periodontal tissue [23]. Hence, they could be used in the treatment of PD. Still, despite its qualities and favorable results, both *in vitro* and *in vivo* [24] studies on the use of DPMSCs in humans with PD are limited [18].

So, the objective of this study was to determine the effect of a DPMSC treatment both the clinical improvement and regeneration of periodontic bone tissue and their relationship with the markers of chronic inflammation and oxidative stress of people in the aging process with PD.

## 2. Materials and Methods

### 2.1. Experimental Design

A quasi-experimental study with 22 patients with PD was designed. The subjects' age range was between 55 to 64 years old. All were volunteers, of both sexes, healthy or with controlled chronic diseases during the last 12 months, without osteoporosis or horizontal periodontal bone defects.

The research protocol was approved by the Ethics Committee of the National Autonomous University of Mexico (UNAM), Zaragoza (25/11/SO/3.4.1.), and registered in ISRCTN12831118. This study was performed in accordance with the Declaration of Helsinki, and all participants signed the written informed consent.

After a first screening, 48 patients were excluded from the final sample because they did not meet the inclusion criteria (*n* = 37), declined to participate (*n* = 3), or did not attend the first appointment (*n* = 8). Thus, for this study, 22 patients with periodontitis were finally enrolled.

**Figure 1:**
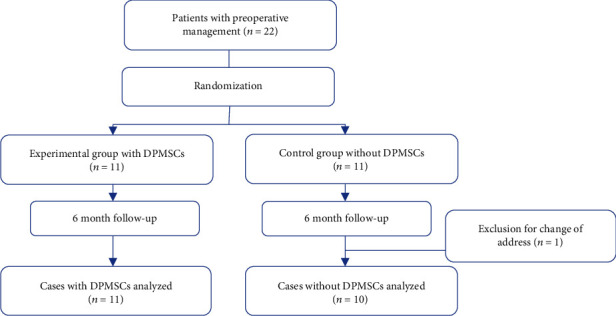
General scheme for study tracking.

The volunteers who met the inclusion and exclusion criteria underwent nonsurgical treatment, in order to control the acute phase of the disease and to manipulate the tissues. This treatment consisted of hygienic-dietary instructions and scaling and root planing. Enrolled subjects were randomly divided into 2 groups (Figure 1). For the experimental group (EG), a collagen scaffold plus 5 × 10^6^ of DPMSCs was placed by periodontal surgery. On the other hand, for the control group (CG), only collagen scaffolding without cells was placed. Both groups underwent probing, mobility, bone mineral density, and saliva sampling to determine the Total Antioxidants Status (TAS), superoxide dismutase (SOD), lipoperoxides (LPO), and interleukins (IL) levels.

### 2.2. Cell Culture

Mesenchymal stem cells were obtained from the dental pulp of three donors (two male patients ages 7 and 8 and a 10-year-old patient) after their parents' signed the written informed consent. All the samples were obtained under aseptic conditions and under the strict criteria of good manufacturing practices, using animal-origin free reagents.

**Figure 2:**
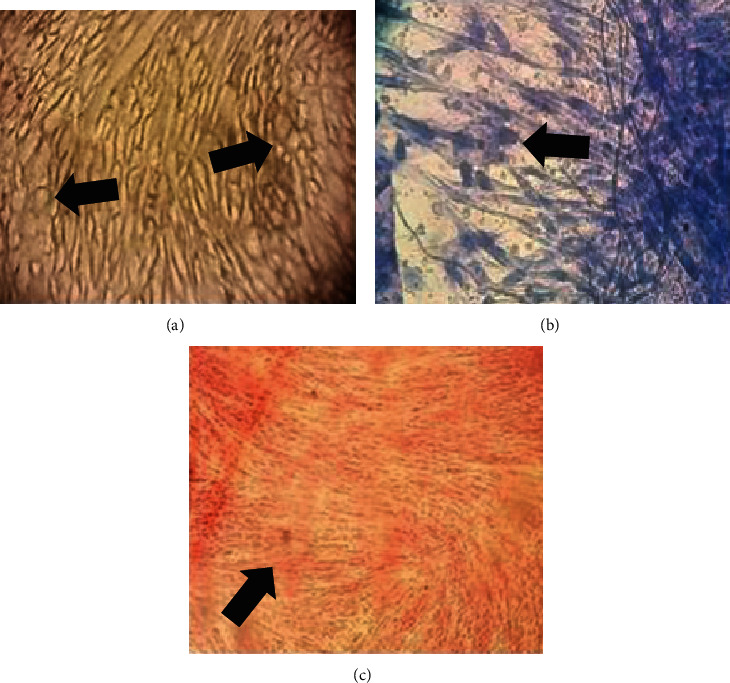
Representative images showing *in vitro* differentiation of multiple mesenchymal stem cell lineages from dental pulp obtained from a 7-year-old donor: (a) Red oil staining showing lipid deposits (arrows), indicative of adipogenic lineage; (b) alcian blue staining showing glycosaminoglycan deposits (arrows), indicative of chondrogenic lineage; and (c) alizarin red staining showing more densely stained areas with mineral deposits (arrows), indicative of osteogenic lineage; (all images, original magnification × 40).

The procedure for all samples was as follows. The dental pulp was gently extracted from the teeth and immersed in a digestive solution (3 mg/ml collagenase type I plus 4 mg/ml dispase in Minimum Essential Medium-Alpha (MEM-*α*) (Life Technologies, Grand Island, New York United States of America) for 1 hour at 37° C. After the dental pulp had been digested, it was dissociated and centrifuged at 497 G for 5 minutes. After centrifugation, it was resuspended in MEM-*α* and incubated at 37° C until 80% confluence was reached. The cells were analyzed by flow cytometry, and differentiation tests were performed for osteogenic, adipogenic, and chondrogenic lineages (Figure 2). The present findings were consistent with the criteria of the International Society of Cell Therapy for mesenchymal stem cells [25].

### 2.3. Treatment

**Figure 3:**
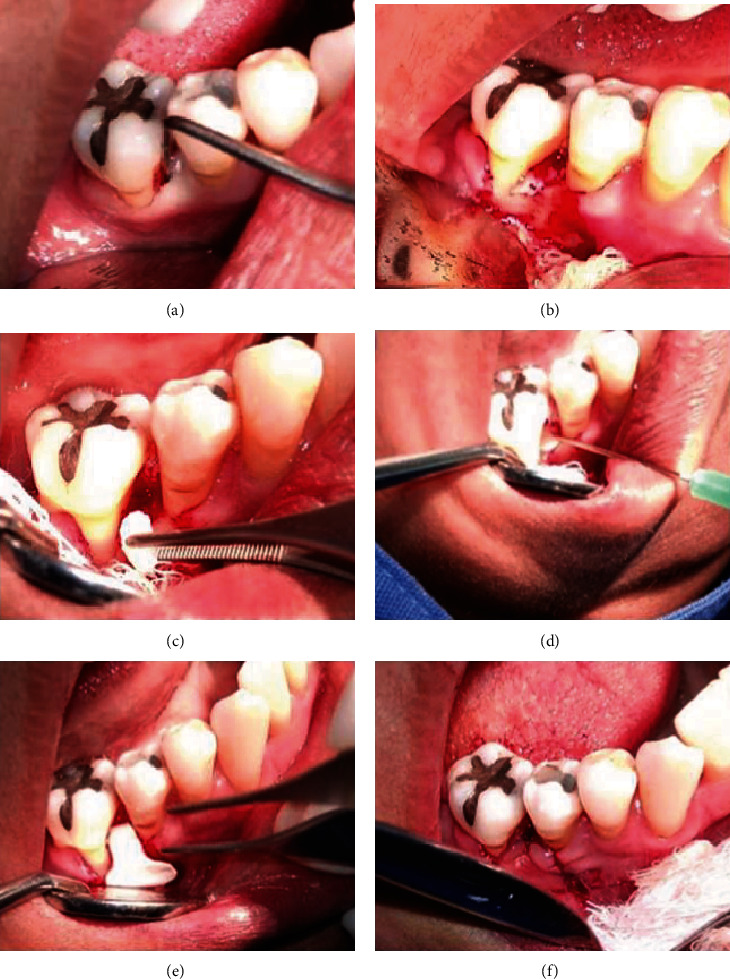
Representative images showing the intervention. (a) Periodontal defect is shown; (b) exposure of the periodontal defect before treatment; (c) placement of the collagen scaffolding; (d) placement of the DPMSCs; (e) placement of the membrane; and (f) sutured flap.

Before the intervention, rinses were indicated for 2 minutes with 0.12% chlorhexidine. After performing asepsis of the area with benzalkonium chloride, locoregional anesthesia was applied through lidocaine and adrenaline infiltrations 1 : 50,000 to continue surgical access in the area of the periodontal bone defect using a flap technique, which consisted of in making an initial incision parallel to the longitudinal axis of the teeth, with a scalloped design and vertical discharge incisions. Then, a mucoperiosteal flap was raised, which was necessary to allow access to the root and bone surfaces. The root surfaces were smoothed with an ultrasonic reamer, the granulation tissue was peeled off, and the area was irrigated with saline water. Once the surface was cleaned, the bone defect was filled with lyophilized polyvinylpyrrolidone sponge® (clg-PVP) in 0.5 cm^2^ fragments with an Adson forceps soaked in chlorhexidine gel. Subsequently, the EG patients had 5 × 10^6^ DPMSCs dripped suspended in 200 ml of PBS, while the CG only received 200 ml of PBS without DPMSCs. Finally, in both groups, collagen membranes (Biomed extend®) were placed and the flap was sutured with Viacryl®. A drop of periacril was placed, and finally Coe-pack® was added to the area to protect the wound (Figure 3).

To control pain and edema, 100 mg nimesulide was indicated at the end of the intervention and 12 hours later. Finally, the stability of the patient was ensured, and care instructions were given. The need to control the biofilm in the areas during the first 2 weeks was explained to the patient by means of rinsing with chlorhexidine solution for 2 minutes twice a day; the use of Tebodont® toothpaste and dental cleaning with the curaprox surgical brush®. Weekly check-ups were scheduled to monitor the healing process. Surgical cement was removed after 7 days, and a tomography was performed 3 and 6 months after surgery.

### 2.4. Statistical Analysis

The data was analyzed using descriptive statistics, where we determined the mean and standard deviation (SD) and perform the Mann Whitney *U* test. A value of <0.05 is considered statistically significant. Values were determined using the SPSS statistical analysis program, version 20.0.

## 3. Results

### 3.1. General

Sociodemographic characteristics and teeth were involved in the study are presented in Table 1.

### 3.2. Effect of DPMSCs on Clinical Parameters

In Table 2, we show that both the group to which collagen plus DPMSCs was placed and the group to which only collagen was placed demonstrated an increase in bone mineral density; however, the increase in the DPMSCs group is almost twice more than the group without cells (with DPMSCs, 142.442 ± 19.5 vs. without DPMSCs, 50.262 ± 9.1 HU) with a borderline statistical significance (*p* = 0.09). Similarly, regarding the depth of periodontal defect (DPD), a statistically significant clinical improvement was observed in the group with DPMSCs compared to the group with no cells placed (with DPMSCs, −3.32 ± 0.12 vs. without DPMSCs, −1.80 ± 0.15 mm, *p* < 0.001).

Regarding the degree of dental mobility, 100% of the patients in the group with DPMSCs and in the group without DPMSCs stopped having grade III mobility; nevertheless, 73% of the first group changed from grade III to grade I, in contrast to 40% observed in the group that did not have DPMSCs grafts (Table 2).

**Figure 4:**
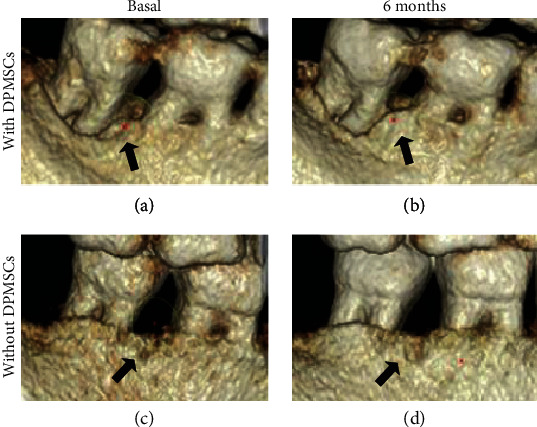
Cone beam volumetric tomography and radiography in which the growth of bone tissue is observed in the EG with DPMSCs (a, b) and the CG without DPMSCs (c, d).

On the other hand, Figure 4 shows the tomographic and radiological images of a case of a patient in the group with DPMSCs and another in the group without DPMSCs, where a bone type filler was more clinically evident in the first group compared to the second was observed. In other words, the quantitative results agree with what was observed in the tomographies, where the presence of radiopaque tissue in the area of the periodontal defect is seen (Figure 3).

### 3.3. Effect of DPMSC on Oxidative Stress Levels

Regarding the oxidative stress markers in saliva, it was observed that after treatment with DPMSCs, there is a statistically significant increase in SOD (baseline, 1.49 ± 0.96 vs. posttreatment, 2.14 ± 1.12 U/L, *p* < 0.05) in the group with DPMSCs (Table 3).

### 3.4. Effect of DPMSC on Levels of Proinflammatory Interleukins

Likewise, in relation to the effect of DPMSCs on the concentration of interleukins IL-1*β*, IL-6, IL-8, IL-10, and TNF-*α*, it was found that the group to which DPMSCs were placed had a decrease in IL-1*β* (baseline, 1001.91 ± 675.53 vs. posttreatment, 722.30 ± 349.45 pg/ml, *p* < 0.05) and that IL-6 also increased when grafting DPMSCs (baseline, 11.31 ± 5.76 vs. posttreatment, 28.06 ± 18.43 pg/ml, *p* = 0.06) (Table 4).

Finally, during the recovery period, the patients had no complications, only minimal pain controlled with pain relievers.

## 4. Discussion

Periodontal disease is a consequence of an exacerbated immune response generated by the body to the excessive accumulation of Gram-negative bacteria [26]. The direct or indirect degradation of the collagen of the extracellular matrix collagen in PD is the result of the activation of proteases and phagocytosis. Furthermore, the release of proinflammatory interleukins is also disproportionately stimulated through NF-*κ*B activation and PG-E2 production by lipid peroxidation and superoxide release, which is related to bone resorption [27].

In order to treat PD, several treatments have been proposed; yet, scientific community continues to looking for one method that allows the regeneration of the periodontium. Regeneration of periodontal tissues lost through PD is a true challenge because the alveolar bone, cementum, and periodontal ligament must be restored to their original architecture and physiology. For this reason, mesenchymal stem cells (MSCs) allogeneic transplantation such as dental pulp mesenchymal stem cells derived from deciduous teeth (DPMSCs) is a good option for treating PD [28].

DPMSCs have been shown *in vitro* to be able to differentiate into the necessary cells to repair tissues and also target inflamed areas while secreting anti-inflammatory interleukins that modulate the response of the immune system [29]. Conversely, some preclinical trials suggest that MSCs have antioxidant potential and, consequently, the ability to reduce the OxS that occurs during inflammation in PD [30, 31].

In this context, promising results have been found reported *in vitro* [32–34] and in animal models [14–17, 35–43]; even so, human research to support clinical application is limited [44–47].

Human studies have about DPMSC and PD are generally been conducted in young patients. However, the problem is that PD occurs mainly in people over the age of 40, in which most cells, including those of periodontal tissue, go into senescence [28].

Additionally, although DPMSCs have multiple functions, they decrease with age [48]. Thus, performing an autogenous graft, (with cells from the same individual), involves two important limitations: The first one is the ethical complication of extracting the pulp from a healthy tooth trying to rescue another with periodontal damage disease. And secondly, even if the DPMSCs were obtained from the patient, probably his cells would have already gone into senescence due to the changes that take place during the aging process [28].

Therefore, a useful alternative is to opt for MSCs from a young exogenous source or from children, in order to overcome the limitations of aged tissues and repair them. This, altogether with the low immunogenicity of the DPMSCs and its easy obtaining prompted us to choose them to carry out the present study.

Accordingly, we present the results of the effect of DPMSCs on a collagen scaffold for the regeneration of periodontal tissues in aging patients with vertical bone defects caused by PD.

The therapeutic effects related to the regeneration of periodontal tissue were evaluated through radiographic images, clinical parameters (as has been proposed in the clinical setting) [44–47], and also with the measurement of bone mineral density (BMD) through tomography [49]. In this sense, in our study, an increase in BMD twice more was observed in the group with DPMSCs compared to the group without them, with borderline statistical significance.

In this regard, the tissue formation within the bone defect of our patients may be due to the fact that DPMSCs differentiate rapidly in osteoblasts and endotheliocytes [50], which allows the formation of bone and the blood vessels necessary to irrigate the newformed tissue. Besides, it has been observed that the DPMSCs placed in bone defects express bone morphogenic proteins 2 (BMP2), which in turn produce a greater amount of bone, which is later mineralized, as reported by Liu et al. (2011) and Aimetti et al. (2014), who observed by radiographs, that after the placement of an MSCs graft, the defect had been completely filled by a bone-like tissue [51, 52].

The increase in BMD with DPMSCs could be caused by two mechanisms. The first is explained by differentiation and proliferation of DPMSCs from the donor within the recipient's tissues, and the second mechanism is through a complex cellular communication system between grafted DPMSCs and receptor periodontal tissue cells, which are aged and damaged [24]. To complement the images showing new bone formation, it would be useful to determine markers such as BALP, Trap5b, Ntx, Ctx, and MMP-8 that indicate the rate of bone remodelling formation, resorption, and regulation in the long term [53].

Regarding Depth of Periodontal Defect (DPD), in our study, a statistically significant decrease was observed in the group with DPMSCs compared to the group without them. This suggests that the DPMSCs promote cell proliferation resulting in an increase in both the gingival tissue and the underlying one, which causes its insertion to be located more coronally. This finding is consistent with what was previously reported by our research group, in a clinical case [54] and with other studies done by Aimetti et al. (2014), who described a clinical case with a periodontal defect, treated by MSCs obtained from one of the third molars of the same patient, whereas after one year of intervention it was observed that DPD had decreased and the degree of mobility was also reduced [52]. This is due to the fact that both the regeneration of the gingival sulcus epithelium and bone tissue formation, favoring that the epithelial adhesion is more coronally repositioned; thus, reducing DPD as indicated by Sculean et al. (2004), in a study with infraosseous defects in humans [55].

Concerning radiographic analysis, in our study, an increase in the area of the periodontal defect was observed, which is consistent with various preclinical and clinical trials, whose reports were that after the placement of different types of MSCs, a radiopaque area was observed radiographically in the bone defect coronal part [43–47, 54].

On the contrary, MSCs have both enzymatic and nonenzymatic biological mechanisms, which help in neutralizing ROS and correcting the damage caused to the proteome and genome by OxS, thereby, efficient management of OxS is guaranteed [56]. Since OxS is closely related to PD, in our study, we evaluated the concentrations of lipoperoxides, total antioxidants, and also superoxide dismutase (SOD). In this regard, it was observed that 6 months after the placement of the DPMSCs graft, there was a statistically significant increase in the concentration of SOD compared to the group that was not treated with DPMSCs. This is consistent with other studies that have shown that MSCs increase SOD levels, supporting the proposed antioxidant effect of MSCs [57].

On this subject, some studies have shown that MSCs can resist high levels of OxS and its induced death. This is related to the reduction in apoptosis and the ability of MSCs to process peroxide and peroxynitrite, since the latter is associated with the activity of SOD1, SOD2, CAT, and GPX1 enzymes in MSCs and a high level of intracellular GSx [30]. Furthermore, MSCs express a high level of methionine and sulfoxide reductase A; the last one acting as an important enzyme for repairing oxidized proteins and for recovering methionine residues that act as preserve the free-radical sink. [58].

Although treatment with MSCs can prevent decreased SOD activity [59], it is important to mention that both the synthesis and the release of SOD by MSCs are synergistically regulated by inflammatory mediators such as TNF-*α* and INF-*γ* preventing damage tissue [60]. In this regard, it has been shown that the correct proliferation, migration, and maturation of DPMSCs depend on the molecules found in the environment and on genetic control [61]. The combined action of signal transduction that is induced by some interleukins determines that DPMSCs differentiate into an osteogenic lineage [62, 63]. Additionally, it is known that high concentrations of proinflammatory interleukins play a fundamental role in the breakdown of periodontal tissue [64].

Although, a genetic expression analysis has proposed the predominant effect of MSCs treatment on the activation of inflammatory pathways “normalizing” inflammation levels [65]. In this regard, in the present investigation, a statistically significant decrease in the levels of IL-1*β* was observed in the group to which the DPMSCs were placed compared to the group without them. This interleukin is closely related to the existence and severity of PD. This statement is in agreement with several studies that affirm that IL-1*β* is synthesized by several cell lines of periodontal tissue and that it occupies a central position among the mediators of the inflammatory cascade during PD [66–68]. It is undeniable that the increase in IL-1*β* production is associated with the development of periodontal disease [69] and that the use of inhibitors of this cytokine contributes to the reduction of periodontal bone loss in primates [70], so it is suggested that the monitoring of IL1*β* levels is a useful approach for determining therapeutic outcomes and a potential target treatments for PD [69, 71]. Therefore, our findings support the hypothesis that not only stem cells reduce the concentration of IL-1*β* but also favor an increase in bone regeneration in patients with periodontal disease.

Having said that, although high levels of IL-1*β* are associated with PD, it is also true that early IL-1*β* effectively induces mesenchymal stem cells to differentiate into osteoblasts and tissue to become mineralized [72]. Consequently, it would be advisable in future studies to carry out a short-term evaluation after the placement of the DPMSCs.

Also, it has been reported that after the administration of MSCs, the inflammatory microenvironment is promoted to an anti-inflammatory one, by inhibiting the production of proinflammatory cytokines (such as TNF-*α* and IL-6) and regulating endothelial permeability. These effects can be mediated by paracrine mechanisms that control the inflammation cascade [73]. IL-6 is involved in the differentiation of DPMSCs towards preosteoblasts [74]. For this reason, it is possible that it intervenes in the regeneration of the periodontium [64, 68]. In this regard, in our study, IL-6 concentrations showed a decrease with a tendency to difference in the group treated with DPMSCs.

On the other hand, in our research, it was observed that the patients treated with DPMSCs had no signs or symptoms of rejection, which is consistent with previous clinical studies that indicate that none of the patients had adverse effects on mesenchymal stem cell grafting, since these are cells have immunosuppressive activity, allowing them to be used allogeneically in the treatment of various diseases [75, 76]. Similarly, our results agree with those of the study by Feng et al. (2010), who observed that patients who underwent periodontal ligament cells had a beneficial effect on periodontal defects and had an uncomplicated healing of the gum during the first 3 weeks [45].

This may be related to the fact that MSCs suppress the proliferation of CD4^+^, CD8^+^, B cells, and natural killer T cells [65]. They also induce the proliferation of CD4/CD25 regulatory T cells (Tregs), when cocultured with blood mononuclear cells, and can activate macrophages influencing them to differentiate into an anti-inflammatory immunophenotype [77–80].

The immunodulatory capacity of MSCs is an important property exploited for the treatment of diseases related to inflammation, such as PD, because they can regulate both the innate and adaptive immune response [81–83].

Some studies suggest that MSCs can inhibit activation of T lymphocytes, natural killer cells, and dendritic cells [84], and that type 1 dendritic cells are induced by MSCs to reduce TNF-*α* secretion and type dendritic cells 2 to increase IL-10 secretion [85, 86]. Likewise, Th1 cells reduce the secretion of INF-*γ* and Th2 cells increase the secretion of IL-4 [87], which induces a higher immune tolerance a phenotype [88].

MSCs can suppress immune reactions both *in vitro* and *in vivo*[89] because they express low levels of the Major Histocompatibility Complex (MHC) I antigens and do not express MHC II. Accordingly, it is proposed that MSCs have an “immunological privilege” that allows them to be used in allogeneic transplants without problems [90].

Studies with autologous and allogeneic MSCs reveal that these produce soluble factors such as TGF-*β* and hepatocyte growth factor that suppress lymphocyte proliferation [91]. In incipient clinical trials where MSCs are used allogeneically, they have been shown to have good tolerance and to not induce an immune response when transplanted into an unrelated receptor [92, 93], as observed in our study. In this sense, preclinical and clinical studies applying MSC reveal beneficial effects to solve different pathologies, but that does not exclude some risks of acute problems (such as immune-mediated reaction and embolic phenomenon), intermediate problems (like graft versus host disease and secondary infection), and long-term problems (as risk of malignancy), due to controversial results on these risks [94], it is recommended to monitor the long-term safety of MSC use, quality control, and clinical-grade production that includes cell viability, endotoxin, and oncogenic assays [95].

Finally, our findings show a therapeutic advantage in the application of mesenchymal stem cells in vertical defects, bone neoformation, antioxidant, and anti-inflammatory activity compared to conventional treatment. Hence, DPMSC treatment could be a more effective therapeutic option for the recovery of connective tissues around the tooth, which have been lost due to PD. Although it is important to point out that one limitation of the study was the reduced sample size, likewise, the groups were not proportionally divided by age and sex, so the influence of these variables could not be evaluated. In addition, the study follow-up was only at 6 months, and the effectiveness of the treatment cannot be assured any longer. In this sense, although the potential risks of MSC administration cannot be completely ruled out in our study, the function of MSCs appears to be mediated through a “hit and run” mechanism rather than through a sustained engraftment in the injured tissues. The low degree of MSC engraftment may limit the long-term advantages of MSC therapy [96]. Also, both the localized issue of periodontal disease and of the therapeutic procedure proposed here could act in favor rather than to the detriment of the patient's health.

## 5. Conclusions

Our findings suggest that a DPMSCs treatment has an effect on periodontal bone regeneration in periodontal disease in aging people, linked to an increased superoxide dismutase, and decreased proinflammatory interleukins. Therefore, we conclude that a DPMSCs treatment can be a useful option to regenerate the lost tissues in periodontal disease.

## Figures and Tables

**Table 1:** Demographic data and characteristics of the study population.

Characteristics	With DPMSCs	Without DPMSCs	Total
No. of subjects, *n*	11	10	21
Age (y), mean ± SD	59.1 ± 5.12	59.7 ± 5.25	59.4 ± 5.19
Gender, *n* (%)			
Female	4 (63.6)	3 (40)	7 (33.3)
Male	7 (36.4)	7 (70)	14 (66.7)
Education (y), mean ± SD	11.72 ± 3.64	12.0 ± 3.71	11.8 ± 3.68
Systemic diseases, *n* (%)			
Yes	3 (27.3)	4 (40)	7 (33.3)
No	8 (72.7)	6 (60)	14 (66.7)
Regular medication, *n* (%)			
Yes	3 (27.3)	4 (40)	7 (33.3)
No	8 (72.7)	6 (60)	14 (66.7)
Teeth involved, *n* (%)			
Maxilla incisor	1 (9)	0 (0)	1 (4.8)
Maxilla canine	0 (0)	0 (0)	0 (0)
Maxilla premolar	0 (0)	1 (1)	1 (4.8)
Maxilla molar	0 (0)	1 (1)	1 (4.8)
Mandible incisor	2 (18)	0 (0)	2 (9.5)
Mandible canine	0 (0)	0 (0)	0 (0)
Mandible premolar	1 (9)	1 (1)	2 (9.5)
Mandible molar	7 (64)	7 (7)	14 (66.6)

Values show mean ± standard deviation. DPMSCs: dental pulp mesenchymal stem cells; EG: experimental group; CG: control group.

**Table 2:** Effect of DPMSCs on clinical parameters by treatment group.

Parameter	With DPMSCs (*n* = 11)			Without DPMSCs (*n* = 10)			*p*
	Basal	6 months	Difference EG	Basal	6 months	Difference CG	
BMD (HU)	638.82 ± 181.7	781.26 ± 162.2	142.442 ± 19.5	620.49 ± 143.5	670.76 ± 134.4	50.262 ± 9.1	0.098
DPD (mm)	5.66 ± 0.41	2.34 ± 0.29^∗^	−3.32 ± 0.12	5.58 ± 0.38	3.78 ± 0.53	−1.80 ± 0.15	0.001
Dental mobility							
						
Grade III (%)	10 (90)	0 (0)		7 (70)	0 (0)		
Grade II (%)	1 (10)	3 (27)		3 (30)	6 (60)		
Grade I (%)	0 (0)	8 (73)		0 (0)	4 (40)		

Values show mean ± standard deviation. Mann-Whitney *U* and Wilcoxon test, ^∗^*p* < 0.05. DPMSCs: dental pulp mesenchymal stem cells; BMD: bone mineral density; HU: Hunsfield units; Depth of Periodontal Defect (DPD). EG: experimental group; CG: control group.

**Table 3:** Oxidative stress markers in saliva prior to DPMSCs placement and 6 months after that.

Parameter	With DPMSCs (*n* = 11)			Without DPMSCs (*n* = 10)			*p*
	Basal	6 months	Difference EG	Basal	6 months	Difference CG	
TAS (mmol/L)	0.598 ± 0.21	0.757 ± 0.32	0.159 ± 0.35	0.591 ± 0.29	0.640 ± 0.49	0.049 ± 0.36	0.618
SOD (U/L)	1.49 ± 0.96	2.14 ± 1.12^∗^	0.647 ± 0.73	1.386 ± 0.70	1.100 ± 0.57	−0.276 ± 0.65	0.047
LPO (*μ*mol/L)	0.0791 ± 0.02	0.0448 ± 0.35	−0.031 ± 0.04	0.0677 ± 0.03	0.0610 ± 0.03	−0.006 ± 0.03	0.225

Values show mean ± standard deviation. Mann-Whitney *U* test, ^∗^*p* < 0.05. DPMSCs: dental pulp mesenchymal stem cells; TAS: Total Antioxidant Status; SOD: superoxide dismutase; LPO: lipoperoxides. EG: experimental group; CG: control group.

**Table 4:** Proinflammatory interleukins concentrations (pg/ml) in saliva samples prior to and after DPMSCs placement.

Parameter	With DPMSCs (*n* = 11)			Without DPMSCs (*n* = 10)			*p*
	Basal	6 months	Difference EG	Basal	6 months	Difference CG	
TNF-*α*	8.91 ± 3.04	7.66 ± 3.37	−1.24 ± 1.41	9.276 ± 5.98	7.80 ± 5.78	−1.28 ± 1.79	0.844
IL-6	11.31 ± 5.76	28.06 ± 18.43	16.74 ± 5.88	20.13 ± 15.57	22.43 ± 18.40	2.30 ± 4.00	0.067
IL-1*β*	1001.91 ± 657.53	722.30 ± 349.45^∗^	−279.61 ± 167.51	1238.18 ± 779.95	1543.92 ± 827.15	305.73 ± 256.11	0.039
IL-10	2.92 ± 0.91	2.80 ± 0.48	−0.112 ± 0.212	2.62 ± 0.61	3.36 ± 0.56	−2.58 ± 0.19	0.268
IL-8	1270.92 ± 670.64	1145.72 ± 805.99	−125.19 ± 364.71	1497.27 ± 1339.83	827.27 ± 632.15	−670.0 ± 326.30	0.417

Values show mean ± standard error. Mann-Whitney *U* test, ^∗^*p* < 0.05. DPMSCs: dental pulp mesenchymal stem cells; IL: interleukin; TNF-*α*: tumor necrosis factor alpha; EG: experimental group; CG: control group.

## Data Availability

The data used to support the findings of this study are available from the corresponding author upon request.
